# Melanoma Peptide MHC Specific TCR Expressing T-Cell Membrane Camouflaged PLGA Nanoparticles for Treatment of Melanoma Skin Cancer

**DOI:** 10.3389/fbioe.2020.00943

**Published:** 2020-08-11

**Authors:** Serkan Yaman, Harish Ramachandramoorthy, Gizem Oter, Daria Zhukova, Tam Nguyen, Manoj K. Sabnani, Jon A. Weidanz, Kytai T. Nguyen

**Affiliations:** ^1^Department of Bioengineering, The University of Texas at Arlington, Arlington, TX, United States; ^2^Joint Bioengineering Program, The University of Texas Southwestern Medical Center, Dallas, TX, United States; ^3^Department of Biology, University of Texas at Arlington, Arlington, TX, United States

**Keywords:** membrane-based drug delivery, T-cell membrane-coated nanoparticles, theragnostic nanoparticles, melanoma, cancer therapy

## Abstract

Melanoma is one of the most aggressive skin cancers, and the American Cancer Society reports that every hour, one person dies from melanoma. While there are a number of treatments currently available for melanoma (e.g., surgery, chemotherapy, immunotherapy, and radiation therapy), they face several problems including inadequate response rates, high toxicity, severe side effects due to non-specific targeting of anti-cancer drugs, and the development of multidrug resistance during prolonged treatment. To improve chemo-drug therapeutic efficiency and overcome these mentioned limitations, a multifunctional nanoparticle has been developed to effectively target and treat melanoma. Specifically, poly (lactic-co-glycolic acid) (PLGA) nanoparticles (NPs) were coated with a cellular membrane derived from the T cell hybridoma, 19LF6 endowed with a melanoma-specific anti-gp100/HLA-A2 T-cell receptor (TCR) and loaded with an FDA-approved melanoma chemotherapeutic drug Trametinib. T-cell membrane camouflaged Trametinib loaded PLGA NPs displayed high stability, hemo- and cyto-compatibility. They also demonstrated membrane coating dependent drug release profiles with the most sustained release from the NPs proportional with the highest amount of membrane used. 19LF6 membrane-coated NPs produced a threefold increase in cellular uptake toward the melanoma cell line *in vitro* compared to that of the bare nanoparticle. Moreover, the binding kinetics and cellular uptake of these particles were shown to be membrane/TCR concentration-dependent. The *in vitro* cancer killing efficiencies of these NPs were significantly higher compared to other NP groups and aligned with binding and uptake characteristics. Particles with the higher membrane content (greater anti-gp100 TCR content) were shown to be more effective when compared to the free drug and negative controls. *In vivo* biodistribution studies displayed the theragnostic capabilities of these NPs with more than a twofold increase in the tumor retention compared to the uncoated and non-specific membrane coated groups. Based on these studies, these T-cell membrane coated NPs emerge as a potential theragnostic carrier for imaging and therapy applications associated with melanoma.

## Graphical Abstract

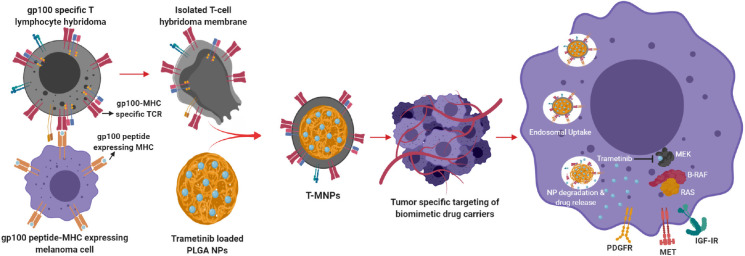

## Introduction

Melanoma is one of the most common cancer types in the United States. It is the most aggressive type of skin cancer and influences people of all ages. Heavy exposure to ultraviolet (UV) radiation, from sunlight or the use of indoor tanning devices (classified as carcinogenic by the International Agency for Research on Cancer) are some of the major causes of melanoma. Risk is also increased for people who are sun-sensitive and those with a weakened immune system (Hayes et al., 2016; Wheatley et al., 2016; Street, 2019). Although many advances have been made for the treatment of aggressive melanoma stages, including improved chemotherapy, targeted therapy, immunotherapy, radiotherapy, and the combinations of the two (Hauschild et al., 2003), most advanced melanoma cases are incurable. Chemotherapy and targeted therapy involve the administration of drugs that often stop DNA transcription or block intracellular signaling pathways in the hope of inhibiting cell growth and replication. In chemotherapy, drugs such as dacarbazine, cisplatin, vinblastine, and temozolomide are widely used. A novel mitogen-activated extracellular signal-regulated kinase (MEK) inhibitor, Trametinib, was approved by the United States Food and Drug Administration (FDA) in May 2013 as a single agent for the treatment of BRAF V600E/K mutant metastatic melanoma (Lugowska et al., 2015). While there are a wide number of therapies and drug combinations available for the treatment of melanoma, a key limitation in the efficacy of chemotherapy is the severe side effects and the development of multidrug resistance during prolonged treatment. Chemotherapy and the listed traditional treatments, however, are often accompanied with insufficient response rates and numerous severe side effects owing to the low efficacy and non-specific targeting mechanisms of drug delivery (Lugowska et al., 2015).

One particular focus of interest in immunotherapy encompasses technologies such as cell therapy. Immune cells are isolated from immunosuppressed cancer patients and reprogrammed *in vitro* against cancer antigens (specific to the cancer present in the patient). Current research has mostly focused on the modification of dendritic cells and T-cells, with T-cell based cancer immunotherapy having been used effectively in cancer treatment (Tran et al., 2014). T-cells are collected from patient’s own body and engineered to generate specific receptors on their surface. These receptors are referred to as chimeric antigen receptors (CARs), which are a specific type of protein located on tumor cells and provide the T-cells with a way to recognize and kill cancer cells (Zhao and Cao, 2019). Although these methods have been proven to be effective, they are accompanied by several limitations. Along the high costs and complexities of isolation, the long-term presence of re-programmed cells in the body poses some major concerns. The possible risks of long-term survival of re-programmed cells have included the development of autoimmune disease and can subsequently lead to death (Sharpe and Mount, 2015; Charrot and Hallam, 2019; Stoiber et al., 2019).

Nanoparticle-based drug delivery systems have recently gained extensive attention for cancer detection and therapy. Nanoparticles possess several advantages, including increased stability, enhanced carrier capacity, varied feasible methods of administration and the ability to incorporate both hydrophilic and hydrophobic types of drugs (Pinto-Alphandary et al., 2000). Nano-vehicles often serve to protect the drug and can be customized to release the drug in a sustained fashion. Sustained kinetics could lead to enhanced drug bioavailability at the cancer site and reduced toxicity to healthy tissues. Several currently studied, nano-based drug delivery platforms for melanoma include polymeric carriers, liposomes, polymersomes, carbon-based nanoparticles, and protein-based nanoparticles (Cai et al., 2014). Polymer based nanocarriers are widely studied due to their long-term stability and ability to provide a sustained drug release over prolonged periods of time. Current FDA approved examples of polymers include PLGA (polylactic-co-glycolic acid) and PLA (polylactic acid). Even though such polymers are biocompatible, they are still far from complete immune evasion (Danhier et al., 2012). To overcome the limitations of current drug delivery and immunotherapy methods, numerous novel modifications have been studied over the last few decades in the hopes of giving polymeric nano-carriers the ability to escape the immune surveillance. Several of these modifications include PEGylation, plasma-surface modification and lipid-based or cell membrane masking. In addition, with the emergence of nanotechnology, the use of bio/nano-carriers is widely expected to alter the landscape of cancer treatment. For example, cell membrane-based drug delivery applications have raised an interest due to the ability of providing better “stealth” properties for foreign biomaterial-based vehicles to remain unnoticed in the face of immune cells. Several of the stealth functionalities of synthetic and biopolymers are used to enable prolonged pharmacokinetics and improve bio-distribution of the particles.

In this study, we have hypothesized that membrane-coated nanoparticles present a comprehensive evasion strategy against the multi-faceted nature of immune clearance and a cancer cell specificity approach. Cell membrane coated biomimetic nanoparticles have made an impressive contribution to the improvement of cancer therapy (Fang et al., 2014) due to the cell membrane structure and retained cellular antigens. Biomimetic NPs carry special advantages, such as long blood circulation, ligand recognition, immune escape, homotypic targeting, and the ability for a sustained drug delivery (Tan et al., 2015). Recently, cell membrane coated nano-systems with unique features and functions have been tested and proved to have higher circulation time and immune evasion (Parodi et al., 2013; Hu et al., 2015). In this study, we have used a hybridoma T-cell with specific targeting against the melanoma cancer. The hybridoma 19LF6 expressed gp100 antigen-MHC molecule recognizing moiety. This cell membrane would be extracted and coated on PLGA nanoparticles encapsulating dyes (coumarin-6 for *in vitro* studies/lipophilic DiD for *in vivo* studies)/Drug (Trametinib) wherein serving as a highly melanoma specific carrier for diagnosis/treatment, respectively. The presence of anti-gp100 TCR on T-MNPs would specifically target gp100 presenting melanoma cells and enhance cellular uptake of T-MNPs. The chemo-drug, Trametinib, was selected due to its effectiveness against cell lines carrying a V600E BRAF oncogenic mutation acquired by DM-6 and 1520 cancer cell lines. Trametinib is an FDA approved MEK (MEK 1 and 2) that can also be used in combination with other approved anti-melanoma drugs. PLGA polymer was chosen for its excellent properties, including biocompatibility, biodegradability, FDA and European Medicine Agency approval, adaptability to both hydrophobic and hydrophilic drugs, and sustained drug release kinetics. Properties of T-MNPs, including binding affinity with skin cancer cells, cellular uptake, therapeutic efficacy, particle retention, and biodistribution, were evaluated using cell cultures and animal models. Their results were also compared to non-specific membrane coated (A-MNPs and D-MNPs) and naked PLGA NPs (NNPs).

## Materials and Methods

### Materials

Poly (D, L lactide-co-glycolide) (PLGA) (inherent viscosity 50:50 with carboxyl end groups) was purchased from Akina (PolySciTech; West Lafayette, IN, United States). Poly (vinyl alcohol) (PVA, MW 30,000–70,000) and DCM (Dichloromethane) were obtained from Sigma Aldrich (St. Louis, MO, United States). Trametinib, coumarin-6, Tris-HCL, D-glucose, *B*-Mercaptoethanol, Phenylmethylsulfonyl fluoride (PMSF), Protease inhibitor cocktail, Triton^®^ X-100, Dimethyl sulfoxide (DMSO) and RPMI-1640 were also received from Sigma-Aldrich. The Avanti Polar Lipids Mini Extruder Kit was ordered from Avanti Polar Lipids (Alabaster, AL, United States). RIPA buffer was purchased from Alfa Aesar (Haverhill, MA, United States). SDS-PAGE gel, and Mini PVDF transfer pack were obtained from Bio-Rad. TCR β chain (Armenia Hamster IgG) – antibody and its isotype antibody were bought from BD Biosciences. Nuetravidin biotin binding protein, Superblock solution, BCA assay kit and DNAse were obtained from Thermofisher Scientific. Formvar coated copper TEM grids were purchased from Electron Microscopy Sciences. Fetal bovine serum (FBS), 1X trypsin EDTA, Dulbecco’s Modified Eagle’s Medium (DMEM) and penicillin-streptomycin were ordered from Invitrogen. Biotinylated gp100-HLA-A2/B2M (gp100 refold) and biotinylated GIGL peptide-HLA-A^∗^201/B2M complexes were synthesized from Dr. Weidanz’s laboratory. gp100 refold heavy chain and Beta-2-Microglobulin chain were expressed in and harvested from *Escherichia coli* in the form of inclusion bodies. The complex was biotinylated using Avidin Biotinylation kit [Bulk BirA: BirA biotin-protein ligase bulk reaction kit]. gp100-2M (209–217) peptide was received from Genscript. bGIGL complex was synthesized in a similar fashion. DM-6, and 1520 cell lines were obtained from Dr. Weidanz’s lab. T-cell hybridomas 19LF6 and DO11.10 were kind gifts from Dr. Devin Lowes (Texas Tech University Health Sciences Center) and Dr. Philippa Marrack (National Jewish Health, Denver, CO, United States), respectively.

### Synthesis of PLGA Nanoparticles

Poly (lactic-co-glycolic acid) nanoparticles (naked NPs; NNPs) were synthesized via a modified single emulsion (O/W) technique as described previously by Menon et al. (2014). Briefly, 90 mg of PLGA 50:50 and 4.5 mg of Trametinib were dissolved in 3 mL of DCM and sonicated at 30 W for 2 min to allow dispersion of PLGA and Trametinib in the solvent. The resulting solution was added in dropwise to 15 ml of filtered 5% (w/v) PVA solution under stirring conditions. The suspension was then sonicated at 30 W for 10 min and then allowed to stir overnight to evaporate the organic solvent. The obtained nanoparticle suspension was centrifuged at 15,000 rpm for 30 min. The supernatant was saved for the drug loading evaluation, and the PLGA NP pellet was re-suspended in 3 ml of DI water followed by freeze-drying for 24 h. Nanoparticles for imaging/diagnostic techniques were synthesized by a similar procedure with coumarin-6 instead of the Trametinib.

### Cell Lines and Culture Conditions

The cell lines used for the experiments, DM-6, 1520, A549 and 19LF6, were maintained in RPMI-1640, supplemented with 10% (v/v) fetal bovine serum (FBS) and 1% (v/v) penicillin streptomycin. DO11.10 and HDF cell lines were maintained in high glucose DMEM, supplemented with 10% (v/v) fetal bovine serum (FBS) and 1% (v/v) penicillin streptomycin. All cells were incubated at 37°C, 5% CO_2_.

### Synthesis of Cell Membrane-Coated NPs (MNPs)

To harvest the cell membrane, cells were grown to confluency in T-225 cell culture flasks. Cells were isolated by trypsinization and centrifuged at 1,000 rpm for 5 min. To remove any remaining media contents, the cells were washed with cold 1X PBS and centrifuged at 1,000 g for 10 min. The resulting pellet was re-suspended in hypotonic lysis buffer (10 mm Tris-HCL, pH = 7.5) and supplemented with the ready-to-use 1× protease inhibitor cocktail. The solution was kept on ice for 20 min and then centrifuged at 1,000 *g* for 10 min. The pellet was re-suspended in cold 0.25 × PBS and kept on ice for 20 min followed by centrifugation at 800 *g* for 5 min. The final pellet was collected in cold 1× PBS. The cell membrane mix was analyzed for the DNA and protein content using Nano-Drop 1000 Spectrophotometer (Thermo Fisher Scientific)/PicoGreen DNA assays and BCA assay kits, respectively. To eliminate any remaining DNA contents, DNAse reaction was performed on the cell extract by incubation with DNAase (the amount of DNAse added varied with the DNA content in the cell extract sample) for least 1 h at 37°C.

Poly (lactic-co-glycolic acid) (PLGA) nanoparticles (naked NPs; NNPs) were coated with different cell membranes (MNPs) including 19LF6, DO11.10 and A549 by adapting a previously used technique by Hu et al. (2011). These particles were loaded with an FDA-approved chemotherapeutic drug, Trametinib, suitable for treatment of melanoma cell lines containing V600E BRAF mutation. Briefly, NNPs were re-suspended in a cell membrane solution at different NNP weight to membrane protein weight ratios (w/w): 1:0.5, 1:1, 1:2 and 1:3. The mixture was then extruded 15 times using a pre-heated Avanti Polar Lipids Mini Extruder (37°C). The extrusion was performed using a 200-nm polycarbonate membrane. The resulting membrane coated NPs (MNPs) were dialyzed with a 100 kDa MWCO dialysis membrane for 2 h. Prior to freeze-drying, dialyzed MNP solution was supplemented with D-glucose at a final concentration of 1 mg/ml. Three types of membrane-coated NPs were created namely: (1) 19LF6 cell line (T-cells specific to melanoma) coated NPs (T-MNPs) – our treatment, (2) DO11.10 cell line (non-specific T-cells) coated NPs (D-MNPs) – control for T-cells, and (3) A549 cell line (lung cancer) coated NPs (A-MNPs) – control for other cell types.

### Physiochemical Characterizations

Nanoparticle size, polydispersity index, and zeta potential were investigated using Dynamic Light Scattering (DLS) technique. To measure the size of the nanoparticles, NP suspension (10 μl of 500 μg/ml) was added to 3 ml of deionized water and inserted into the DLS in a compatible cuvette. To generate TEM images of MNPs, 10 μl of 250 μg/ml MNP suspension was added to plasma treated Formvar Square Mesh Copper Grids and airdried. An H-7500 TEM (Hitachi) transmission electron microscope was used to visualize the particle morphologies. Confirmation of T-cell receptor membrane protein (TCR) on the 19LF6 cell membranes coated nanoparticles was performed via flow cytometry. The particles were also stained with TCR- β chain to evaluate the presence of any TCR or TCR components on synthesized T-MNPs. Briefly, 1mg/ml of T-MNP solution was prepared in a staining buffer (0.5% BSA, 1mm EDTA in 1× PBS). 200 μl of the solutions was added with antibodies Armenian Hamster anti-TCR β and Armenian Hamster IgG antibodies at 1:100 dilution, for test group and isotype control, respectively. The solutions were then incubated for 30 min and then washed 3× times with the staining solution. The cells were collected by centrifuging at 14,000 rpm for 20 min and resuspending in 200 μl of staining buffer. Each of the groups was then analyzed using a BD Biosciences LSR II Flow cytometer without an FSC threshold to be able to detect nanoparticles.

Stability of T-MNPs with varying NP weight to membrane protein weight ratios (w/w) was evaluated by monitoring the particle size at pre-determined time-points using DLS. To observe the stability of T-MNPs, particles of different membrane ratios were incubated in 0.9% saline over 48 h. T-MNP suspensions were prepared as described above, and the size of particles was measured at different time points (0, 0.5, 1, 3, 6, 12, 24, and 48 h).

### Binding Kinetics Assay

The binding characteristics of T-MNPs were studied using ResoSens label-free optical detection system. Biotinylated pMHC complexes HLA-A^∗^02:01–IMDQVPFSV (gp100_209__–__217_) and HLA-A^∗^02:01–GILGFVFTL (Influenza-M, negative control) were synthesized by our group previously. In this study, T-MNPs with the highest and lowest NP weight to membrane weight ratios were tested at varying concentrations. D-MNPs were tested at the highest NP to membrane protein ratio (w/w) (1:2), and at a concentration 2× higher than the highest concentration used for T-MNPs. All samples were tested against a specific gp100-b monomer and non-specific b monomer GILG.

### Drug Loading and Drug Release Kinetics of T-MNPs

The drug/dye loading efficiency was calculated by an indirect method where the drug/dye present in the supernatant collected from the nanoparticle synthesis process was measured, and the following formula was used for loading efficiency calculation:

$$\%\mathrm{loading}\mathrm{efficiency}=\frac{\begin{array}{c} \mathrm{Amount}\,\mathrm{of}\,\mathrm{drug}\,\mathrm{used}-\\ \mathrm{Amount}\,\mathrm{of}\,\mathrm{drug}\,\mathrm{in}\,\mathrm{supernatant} \end{array}}{\mathrm{Amount}\,\mathrm{of}\,\mathrm{drug}\,\mathrm{used}}\times 100$$

The drug (Trametinib) release study was carried out for a period of 28 days. Briefly, 1 mg of NNPs and T-MNPs [NP: membrane protein weight (w/w) ratios of 1:0.5, 1:1 and 1:2] were taken at a concentration of 1 mg/ml and were incubated at 37°C. At each pre-determined time-point, the samples were centrifuged at 14,000 rpm for 30 min, and supernatants were collected and stored at −20°C for later analysis. The pellets were re-suspended in fresh 1× PBS and incubated for further time points. Each of the drug release aliquots was analyzed using a UV-vis Spectrophotometer at 330 nm. The amount of drug released was determined against a standard curve for Trametinib.

### *In vitro* Studies of T-MNPs

#### Assessment of Glycoprotein Expression (gp100) in Tumor Cells by Western Blot and RT PCR Analysis

The expression of glycoprotein (gp100) expression in melanoma cell lines, DM6 and 1520, and lung carcinoma cells A549 were evaluated by western blotting and RT (reverse transcriptase)-PCR. Cellular proteins were extracted from cancer cells using RIPA lysis buffer containing protease cocktail, and protein concentration was measured using the Pierce BCA protein assay kit (#23227, Thermo Fisher, United States). Cellular proteins (12 and 6 μg from each cell line) were separated by 10% SDS-PAGE and electrophoretically transferred onto PVDF membranes. The membranes were blocked with 5% BSA in TBST for 1 h at room temperature, followed by incubation with primary antibodies overnight at 4°C. The membranes were washed with TBST 3× for 5 min and then incubated with HRP conjugated secondary antibodies for 1 h at room temperature. Protein bands were developed using an ECL detection system and imaged using the Chemidoc TM Imaging system.

Total RNA was extracted from DM6, 1520, and A549 cells by using RNeasy plus Mini kits (Qiagen) according to the manufacturer’s instructions. Approximately 3 μg of total RNA was reverse transcribed into cDNA (High-Capacity cDNA RT Kit) and subjected to PCR in Bio-Rad Thermocycler (T100TM) with the following conditions, pre-denaturation at 95°C for 1 min and 30 cycles of denaturation at 95°C for 15 s, primer annealing at 57°C for 30 s, and extension at 72°C for 60 s. The gp100 gene was amplified using the following primers: 5′ GCTTGGTGTCTCAAGGCAACT 3′ (gp100 for) and 5′ CTCCAGGTAAGTATGAGTGAC 3′ (gp100 rev). β-actin gene was amplified using 5′ GGCACCACACCTTCTACAAT 3′ and 5′ GCCTGGATAGCAACGTACAT 3′. The relative amount of each gene was normalized to the amount of β-actin and the RT-PCR result for each gene was expressed as fold change over the basal level.

#### Cellular Uptake and Therapeutic *in vitro* Studies

To determine the cell specific targeting function of the nanoparticle *in vitro*, cellular uptake studies were conducted. Briefly, 1520 (gp100 positive), DM-6 (gp100 positive) and A549 (gp100 negative) cell lines were seeded in a 96 well plate at a density of 20,000 cells/well. Coumarin-6 (C-6; fluorescent dye) loaded T-MNPs (gp100 refold specific) and D-MNPs (non-specific to gp100 refold) at varying NP to membrane weight ratios: 1:0.5, 1:1 and 1:2, were tested. Serially diluted MNP concentrations (100, 250, 500, and 1000 μg/ml) at different NP to membrane ratios were prepared. The cells were exposed to different NP groups for approximately 2 h, subsequently washed with 1× PBS and lysed with 250 μl/well of 1% Triton^®^ X-100. Cell lysis extracts were then analyzed for the protein content using the Pierce BCA protein assay kit (Thermo Scientific, Rockford, IL, United States) and C-6 fluorescent intensity using a UV-vis Spectrophotometer (458/540 ex/em). Total protein concentration in each lysate was determined using a BSA standard curve. The uptake of the nanoparticles was calculated by normalizing the particle concentration (determined from fluorescence intensity in a lysate) in each sample with total cell protein, which correlated to the number of cells in the sample. Untreated cells were used as a negative control.

For the therapeutic efficiency of Trametinib loaded T-MNPs, melanoma cell lines, DM-6 and 1520, were used. Firstly, the effective concentration (IC50) of the Trametinib on the cell lines were determined by exposing cells seeded in a 96 well plate at density of 5,000 cells/well to a free drug of known concentrations (1.2, 2.5, 5, 10, 15, 20, 25, 50, 75, and 100 μg/mL) for a period of 72 h. After 72 h of incubation, the cell death was analyzed using MTS assays (CellTiter 96^®^ AQueous Non-Radioactive Cell Proliferation Assay, Promega) according to manufacturer instructions. Therapeutic efficiency of different nanoparticle groups including 1:2 NP weight to membrane weight ratio T-MNPs (melanoma-specific), A-MNPs (non-specific), and D-MNPs (non-specific) as well as free drug and NNPs (bare/naked NPs) was determined. Three different NP concentrations (0.83, 1.66, and 2.5 μg/ml for DM-6; 15, 29, and 44 μg/ml for 1520) were used based on the calculation of the IC50 studies. Cells seeded in a 96 well plate at a density of 20,000 cells/well were exposed to the described groups for 72 h, and the cell viability was determined using MTS assays.

### Cyto-Compatibility and Hemo-Compatibility

A cyto-compatibility study was performed on human dermal fibroblast cells (HDFs). T-MNPs and NNPs were re-suspended in media and added to cells at various concentrations (50, 100, 250, 500, and 1,000 μg/ml) followed by 24 h of incubation at 37°C. After incubation, cell viability was evaluated using MTS assays. Cells without treatment and cells treated with 1% Triton^®^ X-100 served as positive and negative controls, respectively.

Blood clotting and hemolysis property of T-MNPs were performed on fresh human blood. Briefly, for hemolysis, 200 μl of blood was added with 10 μl of the T-MNPs prepared in 0.9% saline at varying concentrations (50, 100, 250, 500, and 1,000 μg/ml). 10 μl of 0.9% saline and 10 μl of DI water was used as positive and negative controls. The tubes were incubated at 37°C for 2 h under gentle agitation and then were centrifuged at 1,000 *g* for 10 min. The absorbance of sample supernatants was monitored at 545 nm using a UV-vis Spectrophotometer. For hemo-compatibility, 10 μl of T-MNPs at the various concentrations (50, 100, 250, 500, and 1,000 μg/ml) in 0.9% saline was mixed in 50 μl activated blood (0.1 M CaCI_2_ added blood). At pre-determined time-points (10, 20, 30, and 60 min), 1.5 ml of DI water was added to all samples to inactivate blood clotting, and the samples were incubated for 5 min at room temperature. Supernatants were collected and monitored at 540 nm using a UV-vis Spectrophotometer. 0.9% saline and DI water were used as positive and negative controls.

### *In vivo* Biodistribution Studies of T-MNPs via Near-Infrared Fluorescence Imaging

The tumor specific targeting and accumulation of T-MNPs were assessed using a subcutaneous xenograft model of melanoma. It was established by injecting 0.5 × 10^6^ DM-6 cells in 150 μl of Matrigel (Corning, United States) subcutaneously in athymic nude mice. Tumor volumes were checked at regular intervals and treatment started when the volume reached ∼150 mm^3^. For imaging of NP biodistribution *in vivo*, lipophilic DiD (Thermo, United States) dye was incubated with 19LF6 T-MNPs to stain membranes for 30 min and unbound dye was washed away. All nanoparticle groups were injected intravenously through the tail vein. Following, anesthetized mice were scanned at different time points using a Kodak^TM^ *in vivo* imaging system. After 24 h time point, the mice were euthanized under anesthesia. Tumors and organs were excised and prepared for *ex vivo* imaging and fluorescent analysis. Excised organs and tumors then were analyzed by their weights and fluorescent responses.

### Statistical Analysis

All results were expressed as mean ± SD performed with *n* = 3 for most of the experiments if not specified. Results were analyzed using either one-way or two-way ANOVA depending on experiments with *p* < 0.05, and the student’s *t*-test was used to identify differences between groups. *P* < 0.05 was considered to be statistically significant.

## Results

### Particle Size, Zeta Potential, and Morphology

**FIGURE 1:**
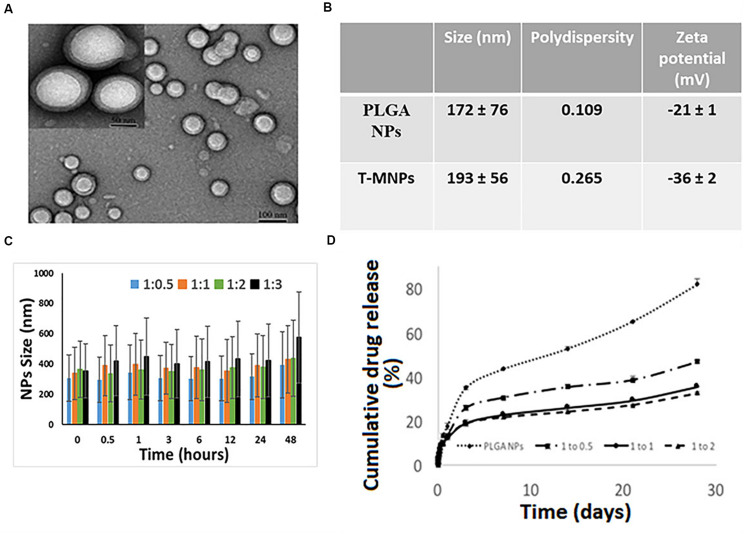
Characterization of T-MNPs. **(A)** TEM images of T-MNPs at 1:2 NP to membrane protein weight ratio. **(B)** Size, polydispersity and zeta potential of PLGA NPs and T-MNPs analyzed using Dynamic Light Scattering (DLS). **(C)** Stability of T-MNPs in 0.9% saline (at varying NP to membrane protein weight ratios: 1:0.5, 1:1, 1:2, and 1:3) evaluated by change in NPs size over a 2-day period using DLS. **(D)** Cumulative% drug release from T-MNPs (varying NP to membrane protein ratios of 1:0.5, 1:1, 1:2 versus PLGA NPs) performed in phosphate buffered saline over 28 days. Samples were analyzed using UV-vis spectrophotometer.

The DLS results showed that the T-MNPs with 1:2 NP to membrane ratio had a particle size of about 193 ± 56 nm with a polydispersity of 0.265. The particle size of naked NPs (NNPs) was around 171.7 ± 76 nm with a polydispersity of 0.109. The particle sizes were confirmed with Transmission Electron Microscopy (Figure 1A). Furthermore, TEM images of T-MNPs showed that the particles were smooth and spherical with a clear core-shell structure. The T-MNPs also had a stable ZETA potential value of −36 mV compared to a −20 mV ZETA potential of the NNPs. The stability studies also showed that the T-MNPs were stable for the whole incubation period, which was denoted by no significant change in the size of the T-MNPs during the course of experiment and, therefore, there was no sign of significant aggregation (Figures 1B,C).

**FIGURE 2:**
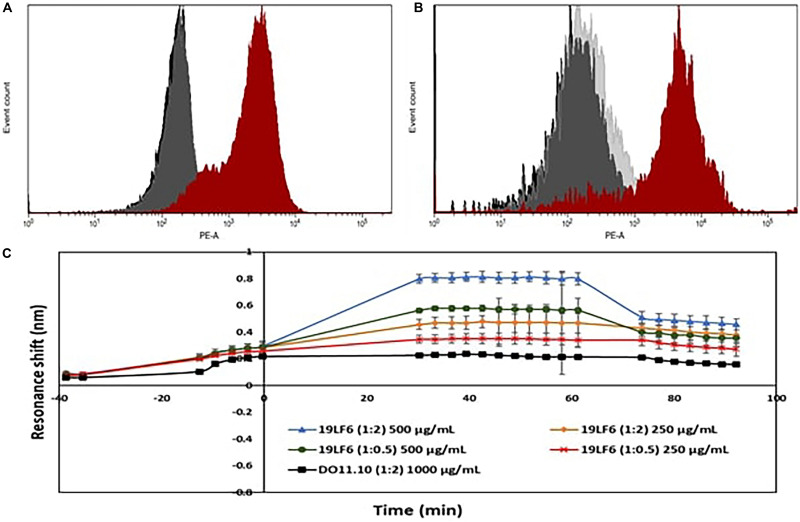
Validation of TCR on T-MNPs. **(A)** Histogram curves demonstrate 19LF6 cells expressing the TCR β chain. 19LF6 cells were stained with either mouse IgG (isotype) or anti-TCR antibody. Unstained, isotype, and stained groups are represented as black, gray, and red curves, respectively. **(B)** T-MNPs also expressed the TCR β chain [19LF6 MNP’s were stained with either mouse IgG (isotype) or anti-TCR antibody]. Unstained, isotype, and stained groups are represented as black, gray, and red curves, respectively. Histograms were analyzed using BD LSR II with no threshold on the forward scatter to detect the nanoparticles. **(C)** Binding kinetics of D-MNPs (non-specific control), T-MNPs (our treatment group) and NNPs (negative control) at varying concentrations and NP to membrane protein (w/w) ratios; kinetics were measured in terms of resonance shift over a period of time using ResoSens label-free optical detection.

Drug loading efficiency of T-MNPs was found to be 61%. To analyze how different membrane coating ratios would affect Trametinib release from T-MNPs, the drug release kinetics of T-MNPs with different NP to membrane weight ratios (1:0.5, 1:1, and 1:2) and naked NPs were evaluated over 28 days. The rate of release kinetics differed across the three ratios of NP to membrane protein (w/w) T-MNPs. The Trametinib release rate was significantly slower and sustained for all ratios of T-MNPs when compared to MNPs, with the lowest rate of release for 1:2 T-MNPs and the highest one for 1:0.5 (Figure 1D). The presence of TCR β chain on 19LF6 cells and T-MNPs were confirmed by flow cytometry analysis as seen by a shift in the absorbance curve (Figures 2A,B).

### Binding Kinetics of T-MNPs

As shown in Figure 2C, T-MNPs bound onto immobilized gp100b in a dose-dependent manner with a higher binding strength observed in samples with higher concentration and higher ratio. In addition, T-MNPs at concentrations 500 and 250 μg/ml were significantly higher when compared to the binding kinetics of 1:2 D-MNPs at 1,000 μg/ml concentration.

### Western Blot and Reverse Transcription PCR

**FIGURE 3:**
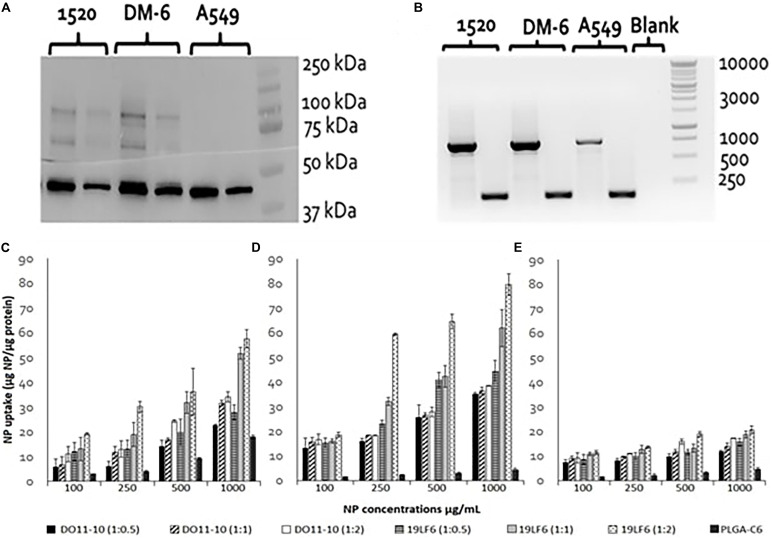
gp100 expression and Cellular uptake of T-MNPs in different cell lines. **(A)** Western blot for assessing of gp100 protein content in 3 cancer cell lines: DM-6, 1520, and A549. β-actin was served as a control (42 kDa). Proteins are represented at two concentrations: 6 μg (right) and 12 μg (left) for each cell line. **(B)** RT-PCR: gp100 (751 bp) expression presented in 1520, DM-6, and A549 cell lines, β-actin served as a control (157 bp). **(C)** DM-6, **(D)** 1520, and **(E)** A549 cell lines were treated with MNPs for 2 h. Cell lysates were analyzed for MNP content using a UV-vis Spectrophotometer and cellular uptake was normalized with total cellular protein. Uptake of D-MNPs, T-MNPs and PLGA NPs (PLGA-C6) are presented at varying NP to membrane protein weight ratios. MNPs were loaded with a fluorescent dye, Coumarin 6 (*n* = 3).

DM6 and 1520 cell lines exhibited significant expression of glycoprotein gp100. The expression level in the 1520 cell line was found to be approximately 2× higher than DM-6, whereas the lung cancer cells A549 showed no apparent gp100 protein expression (Figure 3A). RT-PCR identified gp100 gene amplicon (751 bp) in all 3 cell lines (Figure 3B). The melanoma cancer cell 1520 has demonstrated its higher expression of gp100/HLA-A2 complex protein compared to that of the DM-6 cell line previously (Weidanz et al., 2006).

### *In vitro* Properties of T-MNPs

**FIGURE 4:**
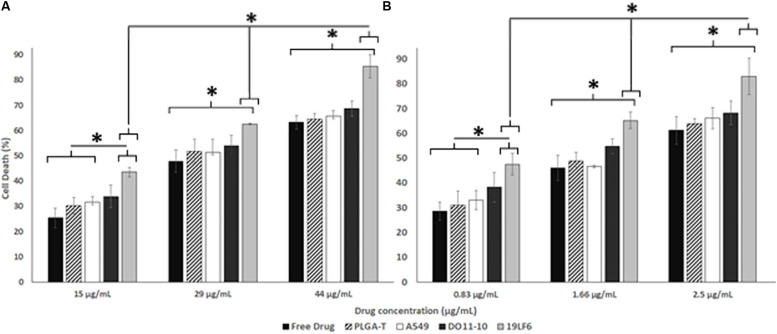
Therapeutic efficiency of T-MNPs. Therapeutic capabilities of T-MNPs, D-MNPs, A-MNPs, NNPs and free Trametinib on **(A)** 1520 melanoma cell line and **(B)** DM-6 melanoma cell lines. Cell were exposed to the nanoparticle suspensions for 72 h (ranging concentrations from IC25 to IC75), and cell viability was evaluated using MTS assays. *Statistically significant with *P* < 0.05.

The cellular uptake of NPs was evaluated on DM-6 (gp100-containing melanoma), 1520 (gp100-containing melanoma), A549 (lung cancer, no gp100 antigen) cell lines. For uptake studies, fluorescent dye (Coumarin 6) loaded NPs were used. To analyze how different NP to membrane weight ratios would affect NP cellular uptake, NPs with 1:0.5, 1:1, and 1:2 ratios were utilized. Such ratios were prepared for D-MNPs and T-MNPs. The results demonstrated that T-MNPs displayed significantly higher uptake compared to the negative control, D-MNPs (Figures 3C–E) in gp100-presenting melanoma cell lines. As expected, A549 did not show any selectivity/cellular uptake toward T-MNPs or D-MNPs. Such result was expected due to the absence of the gp100 antigen on the surface. The IC50 values of Trametinib were calculated to be approximately 29.3 and 1.66 μg/ml for 1520 and DM-6 cell lines, respectively. The therapeutic potential of the Trametinib-loaded T-MNPs was evaluated on both 1520 and DM-6 cell lines. It was shown that T-MNPs had a significantly higher therapeutic efficiency on both melanoma cell lines when compared to the negative controls, specifically at IC50 and IC75 drug concentrations (Figures 4A,B).

### Cyto- and Hemo-Compatibility Studies

**FIGURE 5:**
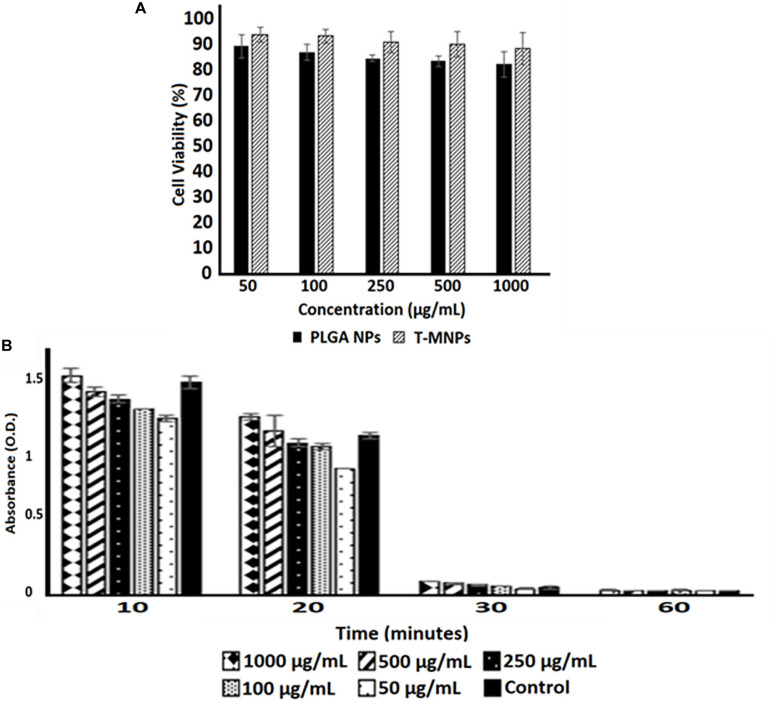
Cyto-/Hemo-compatibility of T-MNPs. **(A)** Cyto-compatibility of T-MNPs was analyzed on human dermal fibroblasts (HDFs) at varying NPs concentrations (50—1,000 μg/ml) for 24 h, and cell viability was quantified using MTS assays (*n* = 3). **(B)** Blood clotting kinetics of T-MNPs. Clotting efficiency was measured in absorbance units (OD) of the supernatants collected from T-MNPs treated blood samples at pre-determined time-points: 10, 20, 30, and 60 min. The absorbances were quantified using a UV-vis Spectrophotometer (*n* = 9).

The viability of HDF cells was evaluated after interaction with the T-MNPs and PLGA NPs (naked NPs, NNPs) at various concentrations for 24 h. Since PLGA is an FDA approved and biocompatible polymer, the cytocompatibility of both NNPs and T-MNPs were also observed in our studies. The result illustrated that T-MNPs did not show any toxicity to the cell line up to a concentration of 1,000 μg/ml, similar to PLGA NPs (Figure 5A). Blood clotting assay was also performed to elucidate potential blood clotting interferences by T-MNPs. The coagulation time of blood in the presence of T-MNPs was examined at different time-points: 10, 20, 30, and 60 min. Blood coagulation initiates an activation of a cascade of coagulation factors and surface mediated reactions (Smith et al., 2015). At all the tested time points, T-MNPs did not display a significantly different blood clotting pattern when compared to that of the saline control (Figure 5B). Furthermore, hemolysis study was performed to test T-MNPs against potential negative effects on red blood cells. T-MNPs showed hemolysis properties lower than 5% up to 1,000 μg/ml concentration.

### *In vivo* Tumor Targeting and Imaging

**FIGURE 6:**
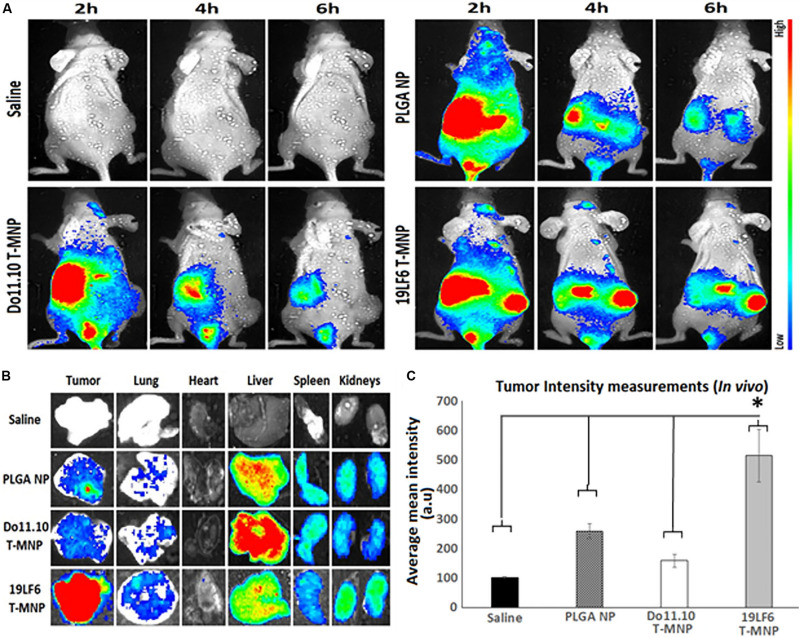
*In vivo* and *ex vivo* analysis of T-MNP biodistribution. **(A)** Real-time tumor targeting characteristics of IV injected NPs on melanoma tumor models. **(B)** *Ex vivo* organ images of biodistribution in different study groups. **(C)** Measured fluorescent intensity of *in vivo* biodistribution study groups in tissue homogenates (*n* = 6 per group). PLGA, poly-lactic-*co*-glycolic acid; NP, nanoparticle; T-MNPs, T-cell membrane-coated PLGA NPs. D-MNP’s, DO10.11 membrane coated PLGA NPs; C-6, coumarin-6; A-MNP’s, A549 membrane coated PLGA NPs; NNP, naked nanoparticle; OD, optical density; IC, inhibitory concentration; RT-PCR, reverse transcriptase polymerase chain reaction; UV-Vis, ultraviolet visible; MTS, 3-(4,5-dimethylthiazol-2-yl)-5-(3-carboxymethoxyphenyl)-2-(4-sulfophenyl)-2H-tetrazolium. *Statistically significant with *P* < 0.05.

The *in vivo* tumor targeting and imaging abilities of the T-MNPs were examined to demonstrate whether injected T-MNPs accumulated at the tumor site during the treatment period. *In vivo* imaging results showed that the T-MNPs (melanoma-specific) had better tumor accumulation than those of the control D-MNPs (non-specific) and NNPs or bare PLGA NPs (Figure 6A). The T-MNPs mainly accumulated in the tumor within 6 h. Furthermore, the tumor signal from the T-MNPs remained relatively constant throughout 24 h, while the overall signal of the NNP and D-MNP groups was decreased with time. In addition, *ex vivo* organ images revealed that T-MNPs could efficiently accumulate at tumor tissues and avoid liver accumulation compared to those of D-MNPs and NNPs in a xenograft mouse model as shown in Figure 6B. The accumulation of T-MNPs was more than twice the amount of nanoparticle accumulation of D-MNPs or NNPs. This result is also confirmed with fluorescent intensity measurements from homogenized tumor tissues (Figure 6C). Therefore, *in vivo* biodistribution results showed that the T-MNP’s targeting ability was confirmed via more efficient accumulation than the NNPs and D-MNPs at the tumor sites.

## Discussion

We have thus developed a theragnostic system for effective diagnosis and treatment of melanoma. The developed nanoparticle system offers great advantages compared to the conventional drug delivery systems including enhanced retention in the cancer cells and tumor sites compared to non-targeted systems. These NPs also provide controlled/sustained drug release for up to 28 days and the least/null cytotoxic behavior. The NP drug delivery system helps to reduce the cytotoxic effects of chemotherapy drugs and is predicted to minimize immune reactions as the nanoparticles are coated in endogenous T-cell membranes. The dye loaded nanoparticles will serve as an effective tool to accurately visualize the tumor site, whereas the drug loaded nanoparticles will provide the therapeutic effectiveness to treat melanoma. Thus, our results indicate the potential of our designed T-MNPs for theragnostic application to detect and treat melanoma.

In the last decade, it has been proven that cells can be used as effective drug carriers via either themselves or their membranes that can facilitate payload delivery to desired regions (Stephan et al., 2010; Tan et al., 2015). Previous works have also shown that healthy immune cells (i.e., leukocytes) are able to circulate around the body, migrate into tissues, transport through inflamed tissues and adhere to inflamed vessel walls (Muller, 2013). In this manner, cell membrane surface proteins such as ICAM’s, CCLs, CXCLs, TCR’s, and/or specific CD macromolecules become important to provide an effective drug delivery platform for biomimetic delivery (Thanuja et al., 2018). Recently, studies have shown that cytotoxic T lymphocyte membranes can deliver their payloads to tumor regions; for example, it has been shown that payload delivery can be facilitated through the natural lymphocyte surface proteins such as LFA-1 (Zhang et al., 2017; Huang et al., 2018). Therefore, this study explored the role of target specific cytotoxic T lymphocyte membranes as a drug delivery platform.

The observed shortcomings of several drug/dye-loaded polymeric nanoparticles are their poor stability/increased aggregation, low control over the payload release rate, and rapid clearance from the body. Our results of T-MNPs showed that coating the nanoparticle with cell membranes could overcome these limitations. Physiochemical characterization of synthesized T-MNPs showed excellent colloidal stability in physiological conditions. The increase in stability due to the membrane coating onto the nanoparticles was evident by the stable Zeta potential of the T-MNPs compared to an unstable potential of the NNPs as described by the −30 mV Zeta rule (Kumar and Dixit, 2017). The negative surface charge (−36 mV) of the T-MNPs repel negative charged albumin molecules in the serum, preventing potential aggregations. The stability of T-MNPs in 0.90% saline solution was found to be similar across different NP weight to membrane protein weight (w/w) ratios for up to 2 days (Figure 1C). Fang et al. (2014) reported a similar observation where they showed a higher stability of the coated nanoparticle for up to 15 days across a wide range of NP to membrane ratios (1:0.5 to 1:4). The loading efficiency of T-MNPs was observed to be approximately 61%, and the drug release kinetics displayed an initial burst release followed by a sustained release up to 28 days. When comparing T-MNPs and NNPs, it can be clearly seen that cloaking of drug loaded PLGA nanoparticles with T-cell membranes reduced the drug release rates, especially those coated with higher amounts of the cell membrane. This reduction has also been observed in nanoparticles cloaked with membranes from erythrocytes (Hu et al., 2011) where erythrocyte membrane-coated nanoparticles showed slow drug release compared to uncoated nanoparticles. Hu et al. (2011) attributed this effect to the membrane’s ability to act as a diffusion barrier to provide better sustained drug release, as compared with PEG-based nanoparticles, thereby enhancing the therapeutic efficacy of the drug in acute myeloid leukemia cells. Furthermore, T-MNPs hold the unique characteristics of natural 19LF6 cell membranes thus avoiding clearance and enhancing circulation. Zhong et al. (2013) previously defined the 19LF6 cell line to contain a metastatic melanoma antigen, gp100 (209–217) – specific T-cell receptor. Our flow cytometric analysis and the binding kinetic results not only confirmed the higher presence of the specific TCR on the nanoparticle coating, but also its ability to bind to its specific target.

As part of the toxicological analysis, the T-MNPs, cyto- and hemo-compatibility were analyzed. The T-MNPs were found to be cyto-compatible up to 1,000 μg/ml concentration, which was shown to be at least as cyto-compatible as NNPs or bare PLGA NPs. This was similar to the results observed by Guo et al. (2015), who demonstrated that erythrocyte-membrane coated PLGA nanoparticles possessed a similar cyto-compatibility compared to bare PLGA nanoparticles. This attributes to the cell mimicking characteristic and the slow drug release characteristics of T-MNPs. Blood clotting characteristics of T-MNPs were compared to the saline control and found to have no significant effect on the blood clotting cascade up to 1,000 μg/ml. According to the criterion in the ASTM E2524-08 standard, percent hemolysis >5% is considered toxic to red blood cells (ASTM E2524-08, 2013). T-MNPs-induced blood hemolysis was observed to be <5% up to 500 μg/ml. Although T-MNPs were found to be toxic to red blood cells at a concentration of 1,000 μg/ml, such high concentrations of the particles might not be used for later studies.

We also continued to investigate the *in vitro* characteristics of these membrane coated nanoparticles and their ability as a highly specific vehicle for cancer targeted delivery. In light of past studies that showed bare PLGA NPs have limitations owing to non-specific targeting and result in uncontrolled tissue distribution of the drug (Danhier et al., 2012) and that cell membrane coating improves immune evasion, target specificity and drug efficacy (Hu et al., 2011), we have devised the proposed design of T-MNPs. Fang et al. (2014), devised surface-engineered PLGA NPs with platelet-membrane-derived vesicles since platelet cells have a natural ability to adhere to injured blood vessels as well as circulating pathogens. Such membrane coating provided the particles with natural platelet-like targeting functions. However, there is no data found in the literature for natural specific targeting (i.e., via TCR receptors) abilities of membrane coated drug carriers so far. The data obtained from our *in vitro* cell studies sheds light to the undiscovered potential of cytotoxic T-cell membrane coated nanoparticles as a biomimicking drug delivery vehicle. In our study, anti-gp100 TCR influence on T-MNP cellular uptake was observed in DM-6 and 1520 melanoma cell lines. Since A549 lung cell lines did not show any apparent gp100 presentation, A549 was used as a negative control cell line. Data collected from binding and cellular uptake studies showed that T-MNPs illustrated superior binding and uptake kinetics attributed to their anti-gp100 TCR. The uptake of T-MNPs at all ratios was significantly higher than that of D-MNPs, the negative control nanoparticles (Figure 3). These particles showed selective and effective binding to gp100 carrying melanoma cells when compared to that of D-MNPs made from the negative T-cell control. The uptake of T-MNPs increased with increasing concentrations and was enhanced in the 1520 cell line even more than that of DM-6 cell line. The enhanced 1520 uptake of the T-MNPs might have been due to its ability to better present gp100/HLA-A2 complex protein compared to that of the DM-6 cell line (Weidanz et al., 2006).

Furthermore, therapeutic efficiency of T-MNPs was evaluated. The IC50 values of Trametinib for DM-6 and 1520 were found to be approximately 29 and 1.6 μg/ml, respectively, which were very much similar to the values observed in a previous study by Roller et al. (2016). However, the IC values often depend on numerous human and in-house factors, therefore, they must be performed for each study. T-MNPs were found to be significantly more efficient in killing melanoma cancer cells than any of other groups compared, even much more than free Trametinib at the same concentration, which would attribute to the binding and uptake characteristics of T-MNPs. Particles with a higher membrane content (greater anti-gp100 TCR content) showed to be more effective when compared to that of the lower NP to membrane ratio. These results indicate that proposed membrane coated natural targeting nanoparticles could potentially be used to improve chemotherapeutic efficacy to effectively treat melanoma.

Following the *in vitro* characterizations, the preliminary *in vivo* investigation by the biodistribution study examined whether intravenously injected T-MNPs targeted and recruited/retained at the subcutaneous tumors in the tumor implanted mice. In biodistribution studies, comparing between T-MNPs and other study groups, expected tumor accumulation difference was observed. NPs from almost all groups accumulated mainly at the liver and spleen in 2 h. The T-MNP group showed distinctive accumulation in the tumor region at almost a threefold higher accumulation on the tumor site than D-MNPs and about a twofold increase in accumulation than NNPs. D-MNPs and NNPs, on the other hand, were captured more in the liver. Similarly, Zhang et al. (2017) used non-specific hCTL membranes to target gastric cancer combined with low dose irradiation and observed very similar results in *in vivo* targeting capabilities of hCTL membrane coated nanoparticles. In their study, membrane coated nanoparticles showed a gradual increase in the tumor sites after low dose irradiation exposure. However, they reported that low dose irradiation was helped for these NPs to accumulate on target tissues at later time points of the study. Surprisingly, they were still able to confirm that non-specific hCTL membrane coated nanoparticles accumulated on tumor tissues without LDI and targeting molecule (Zhang et al., 2017). This effect might be due to the natural adhesion molecules like LFA-1 or integrin of the T-cell membrane surface. In our *in vivo* biodistribution study, anti-gp100 TCR decorated T-cell membranes were able to accumulate on target tissues. What is more to the above-mentioned literature results is tumor tissue accumulation of TCR decorated T-MNPs was superior compared to all control groups from the very early time points. All in all, our proposed T-cell membrane coated drug carrier system showed superior *in vitro* and *in vivo* targeting and uptake capabilities. Therefore, such natural biomaterials as engineered cells, bacteria membranes, biocompatible proteins, viral capsids and others in our drug carrier design can take the theragnostic field further than its current capabilities without compromising the effective drug delivery requirements.

Although cell membrane coated nanocarriers have great potential to deliver drugs to the desired location and could be a promising carrier to improve the theragnostic outcomes in treating melanoma, there are a few associated limitations and challenges. For instance, the cell membrane is comprised of a lot of different protein or peptide types, some of them are required for targeting, evading immune response while the other abundant proteins have unknown interactions in the host environment (Klammt and Lillemeier, 2012). Thus, further in-depth immune response and toxicity profiles must be performed for individual membrane proteins. Cell membrane isolation procedures are not robust and are limited to particular laboratory settings which can be a challenge in clinical translation such as isolation and culture of abundant T-cells in a short duration. Therefore, quality control such as maintaining the functional and structural aspects of cell membranes for longer periods needs to be investigated. Future work should include the required studies to address the above-mentioned limitations of the proposed research. Yet a new approach, utilization of T-MNPs that have been formulated using donor cells and investigating their drug delivery potential as a translational medicine application might be beneficial toward personalized cancer therapies in the future.

## Conclusion

Overall, we successfully developed T-cell coated nanoparticle carriers that displayed superior targeting capabilities toward skin cancer cells and that could serve as a potential tool as a theragnostic system to image and treat melanoma. T-MNPs maintained excellent *in vitro* targeting ability and had a biomimicking shell that minimizes toxicity and systemic clearance concerns in the conventional drug carrier designs. The natural targeting molecule TCR receptor on the surface of the T-MNPs was preserved after membrane isolation and synthesis of T-MNPs. *In vitro* assessments of T-MNPs also showed their therapeutic ability as a drug carrier platform. Finally, biodistribution studies showed the *in vivo* targeting abilities of T-MNPs. The cyto-compatibility and natural targeting T-MNPs recruited to the tumor regions and displayed a distinctive accumulation signal. We showed the therapeutic capabilities of T-MNPs including intrinsic targeting, prolonged drug release and therapeutic potential making T-MNPs an excellent biomimicking theragnostic carrier platform for future cancer therapy.

## Data Availability Statement

The raw data supporting the conclusions of this article will be made available by the authors, without undue reservation, to any qualified researcher.

## Ethics Statement

The animal study was reviewed and approved by Julia Kissling, IACUC/IBC Specialist, Office of Regulatory Services, The University of Texas at Arlington, Texas-76010.

## Author Contributions

SY conceived the original idea whereas JW and KN guided in developing the final nanoparticle design and the planned experiments. GO and DZ carried out the synthesis and characterization of designed nanoparticles and performed some *in vitro* experiments. HR cultured and isolated the cell membranes required for coating of nanoparticles used for some *in vitro* experiments and animal studies. SY, HR, TN, and MS designed and performed the *in vivo* (animal) experiments. SY and HR wrote the manuscript. All authors discussed the results and contributed to the final manuscript. JW and KN supervised the work and helped with the editing of the manuscript.

## Conflict of Interest

The authors declare that the research was conducted in the absence of any commercial or financial relationships that could be construed as a potential conflict of interest. The reviewer PR declared a shared affiliation, with no collaboration, with the authors to the handling Editor at the time of review.
